# Prof. Gunn’s Surgical Clinic

**Published:** 1874-02-15

**Authors:** 


PROF. GUNN'S SURGICAL CLINIC.—At the surgical clinic in Rush Medical College, on Saturday, Jan. 31st, Prof. Gunn operated on a case of necrosis of the femur, after the application of the elastic bandage and ligature. The necrosis, extending through the middle and lower third of the femur, had existed for three years. In the after-treatment of these cases, Prof. Gunn first keeps the wound filled with lint for two or three days; a plug of wax is then substituted, which can be removed at each dressing, and any further spicula of bone that may present be removed without the necessity of a second operation. As granulation proceeds from below, the wax is gradually shaved off from the lower edge.

Several other cases of interest were presented by the clinic, and some minor operations performed.

